# DIETARY METHIONINE RESTRICTION STARTED LATE IN LIFE IMPROVES NEUROMUSCULAR FUNCTION AND PROMOTES HEALTHY AGING

**DOI:** 10.1093/geroni/igae098.3650

**Published:** 2024-12-31

**Authors:** Andrey Parkhitko, Ulalume Hernandez Arciga, Ceda Stamenkovic, Shweta Yadav, Gene Ables, Alessandra Sacco, Michael Jurczak, Stacey J Sukoff Rizzo

**Affiliations:** University of Pittsburgh, Pittsburgh, Pennsylvania, United States; University of Pittsburgh, Pittsburgh, Pennsylvania, United States; Sanford Burnham Prebys Medical Discovery Institute, San Diego, California, United States; University of Pittsburgh, Pittsburgh, Pennsylvania, United States; Orentreich Foundation for the Advancement of Science, Inc, Cold Spring, New York, United States; Sanford Burnham Prebys Medical Discovery Institute, San Diego, California, United States; University of Pittsburgh, Pittsburgh, Pennsylvania, United States; University of Pittsburgh, Pittsburgh, Pennsylvania, United States

## Abstract

Methionine metabolism is negatively affected by aging and restricting methionine consumption (MetR) extends lifespan across diverse species. Similarly, levels of enzymes in the tyrosine degradation pathway (TDP) increase with age, the targeting of which extends Drosophila lifespan. However, we have limited information on the effects of either metabolic manipulation when started late in life in mice, and we lack information on the effects of dietary MetR on the aging process in humans. To investigate the efficacy of targeting either methionine metabolism or the TDP for healthspan improvement in advanced age, we initiated long-term dietary MetR or treatment with the TDP inhibitor, nitisinone, in 18-mo male and female C57BL/6J mice. Our data demonstrate multiple healthspan benefits of dietary MetR started late in life with the strongest improvements observed in neuromuscular function, metabolic health, lung function, and frailty. By contrast, we observed no such signal of benefit with nitisinone treatment. Using single-nucleus RNA and ATAC sequencing of muscle tissues, we identified subtype-specific processes and transcription factors activated by dietary MetR. Epigenetic clocks can be used to evaluate longevity interventions across different species including humans. Surprisingly, dietary MetR did not significantly affect the mouse epigenetic clock. An analysis of human samples obtained from a randomized clinical trial (NCT04701346) examining an 8-week dietary MetR intervention also did not reveal a significant MetR-dependent effect on the epigenetic clocks. These findings establish beneficial effects of MetR started late in life and provide translational rationale to develop MetR mimetics as an anti-aging intervention in humans.

